# Author Correction: Ex vivo editing of human hematopoietic stem cells for erythroid expression of therapeutic proteins

**DOI:** 10.1038/s41467-020-18036-0

**Published:** 2020-08-13

**Authors:** Giulia Pavani, Marine Laurent, Anna Fabiano, Erika Cantelli, Aboud Sakkal, Guillaume Corre, Peter J. Lenting, Jean-Paul Concordet, Magali Toueille, Annarita Miccio, Mario Amendola

**Affiliations:** 1 Genethon, 91000 Evry, France; 2 Université Paris-Saclay, Univ Evry, Inserm, Genethon, Integrare Research Unit UMR_S951, 91000 Evry, France; 3 Laboratory of Hemostasis-Inflammation-Thrombosis, UMR_S1176, Inserm, Univ. Paris-Sud, Université Paris-Saclay, 94276 Le Kremlin-Bicêtre, France; 4 National Museum of Natural History, UMR_1154 Inserm, UMR_7196 CNRS, Univ Sorbonne, Paris, France; 5 Université de Paris, Imagine Institute, Laboratory of chromatin and gene regulation during development, INSERM UMR 1163, F-75015 Paris, France

**Keywords:** Targeted gene repair, CRISPR-Cas9 genome editing, Haematopoietic stem cells

Correction to: *Nature Communications* 10.1038/s41467-020-17552-3, published online 29 July 2020.

The original version of this Article contained errors in the author affiliations.

Giulia Pavani, Marine Laurent, Anna Fabiano, Erika Cantelli, Aboud Sakkal, Guillaume Corre and Mario Amendola were incorrectly associated with ‘INTEGRARE, Genethon, UMR_S951 Inserm, Univ Evry, Univ Paris-Saclay, 91002 Evry, France’ instead of the correct ‘Genethon, 91000, Evry, France’ and ‘Université Paris-Saclay, Univ Evry, Inserm, Genethon, Integrare Research Unit UMR_S951, 91000, Evry, France’.

Magali Toueille was incorrectly associated with ‘INTEGRARE, Genethon, UMR_S951 Inserm, Univ Evry, Univ Paris-Saclay, 91002 Evry, France’ instead of the correct ‘Genethon, 91000, Evry, France’.

Annarita Miccio was incorrectly associated with ‘Imagine Institute, UMR_163 INSERM, Paris, France’ and ‘Paris Descartes Univ Sorbonne Paris Cité, Paris, France’ instead of the correct ‘Université de Paris, Imagine Institute, Laboratory of chromatin and gene regulation during development, INSERM UMR 1163, F-75015, Paris, France.’

This has now been corrected in both the PDF and HTML versions of the Article.

