# Supplementary material for: Ex vivo editing of human hematopoietic stem cells for erythroid expression of therapeutic proteins
Source: Nat Commun. 2020 Jul 29;11:3778. doi: 10.1038/s41467-020-17552-3 (PMC7391635; doi:10.1038/s41467-020-17552-3)

Supplementary Information:

Ex vivo editing of human hematopoietic stem cells for erythroid expression of therapeutic proteins

Giulia Pavani[1], Marine Laurent[1], Anna Fabiano[1], Erika Cantelli[1], Aboud Sakkal[1], Guillaume Corre[1], Peter J. Lenting[2], Jean-Paul Concordet[3], Magali Toueille[1], Annarita Miccio[4], Mario Amendola[1*]

1 INTEGRARE, Genethon, UMR_S951 Inserm, Univ Evry, Univ Paris-Saclay, 91002 Evry, France.
2 Laboratory of Hemostasis-Inflammation-Thrombosis, UMR_S 1176 Inserm, Univ. Paris-Sud, Université Paris-Saclay, 94276, Le Kremlin-Bicêtre, France.
3 Museum National D'Histoire Naturelle, UMR_1154 Inserm, UMR_7196 CNRS, Univ Sorbonne, Paris, France.
4 Imagine Institute, UMR_163 INSERM, Paris, France; Paris Descartes, Univ Sorbonne Paris Cite´, Paris, France.
*Corresponding author: mamendola@genethon.fr

Supplementary Figures…………………... page 2
Supplementary Tables................................ page 14
Supplementary Methods............................... page 21

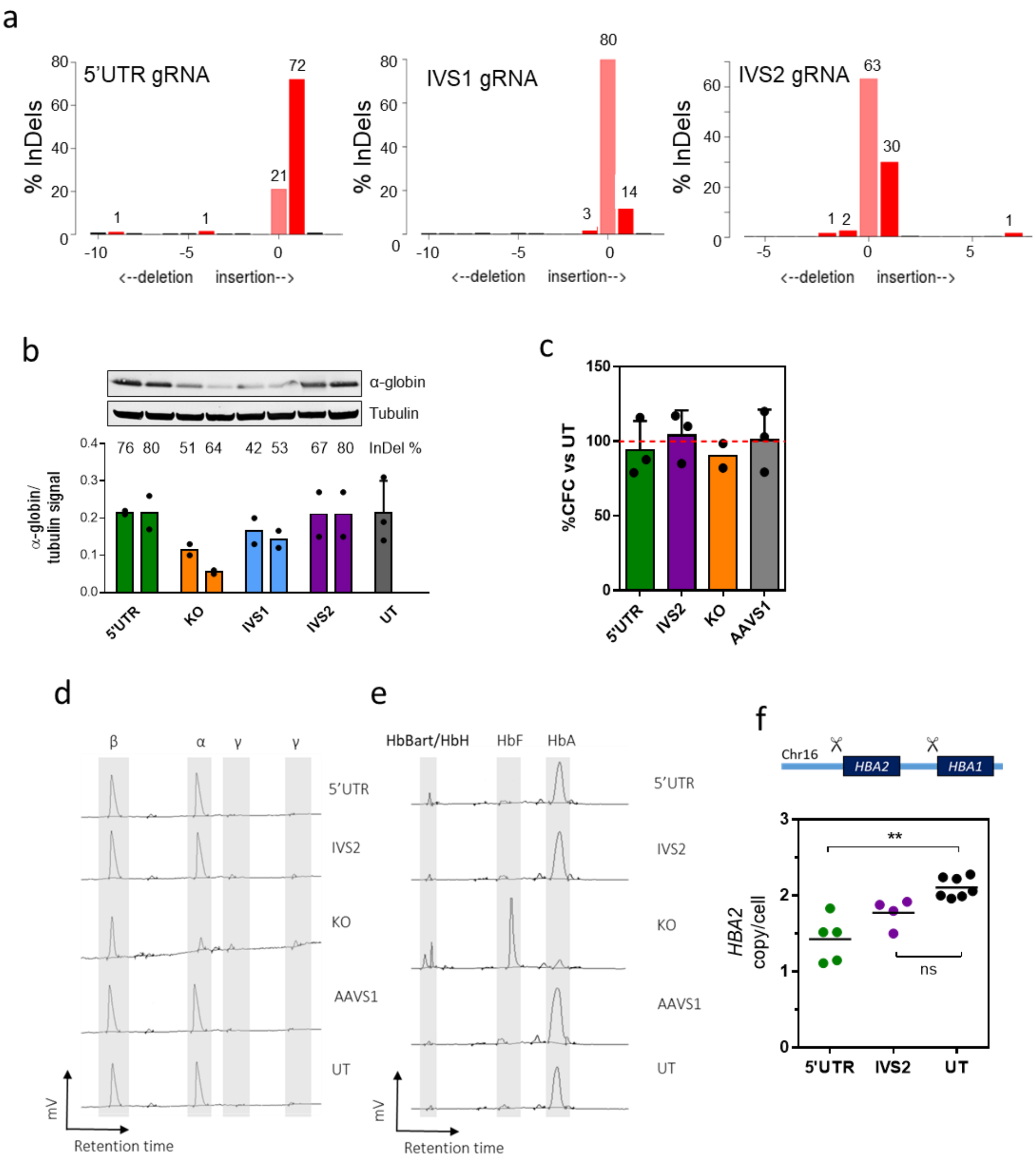

Supplementary Figure 1. (**a**) Representative InDel pattern distribution for 5'UTR, IVS1 and IVS2 HBA gRNAs as assessed by TIDE. (**b**) α-globin expression in K562 cells after genome editing of *HBA*: representative western blot (top) and its quantification (n=2, n=3 for UT; mean ±SD). Percentage of modified *HBA* alleles (indels %) is indicated above bars. Each bar is a different gRNA plasmid transfection, each dot a different analysis. (**c**) CFC numbers expressed as percentage of untreated control (UT). Red dotted line indicates 100%; bars represent mean ±SD (n=3, KO n=2). (**d**) Representative HPLC chromatograms for globin subunit analysis of Figure 1b. (**e**) Representative HPLC chromatograms for globin tetramer analysis of Figure 1d. (**f**) *HBA2* copy number quantification in edited HSPCs by digital droplet PCR (ddPCR) (**, p<0.01; one-way ANOVA, Tukey's test; p=0.008 5'UTR vs UT; n=5 5'UTR, n=4 IVS2, n=7 UT). Schematic representation of *HBA2* deletion is shown above the graph.

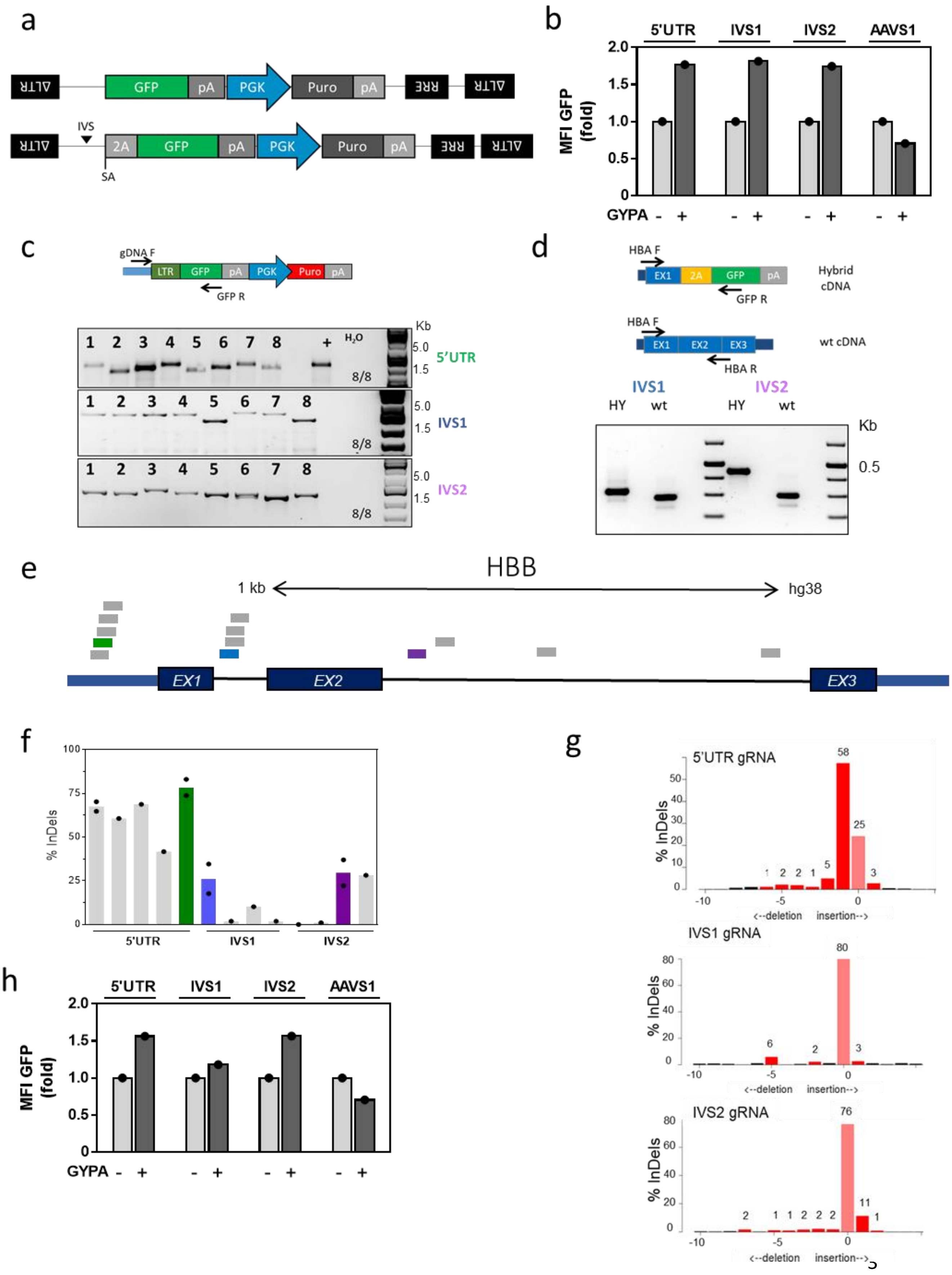
a
ΔLTR
GFP
pA
PGK
Puro
pA
RRE
ΔLTR
IVS
2A
SA
b
5'UTR
IVS1
IVS2
AAVS1
MFI GFP (fold)
GYPA
c
gDNA F
LTR
GFP R
H2O
Kb
5.0
1.5
5'UTR
8/8
IVS1
IVS2
d
HBA F
EX1
2A
Hybrid cDNA
EX2
EX3
wt cDNA
HBA R
HY
wt
0.5
e
HBB
1 kb
hg38
f
% InDels
g
5'UTR gRNA
IVS1 gRNA
IVS2 gRNA
<--deletion
insertion-->
h

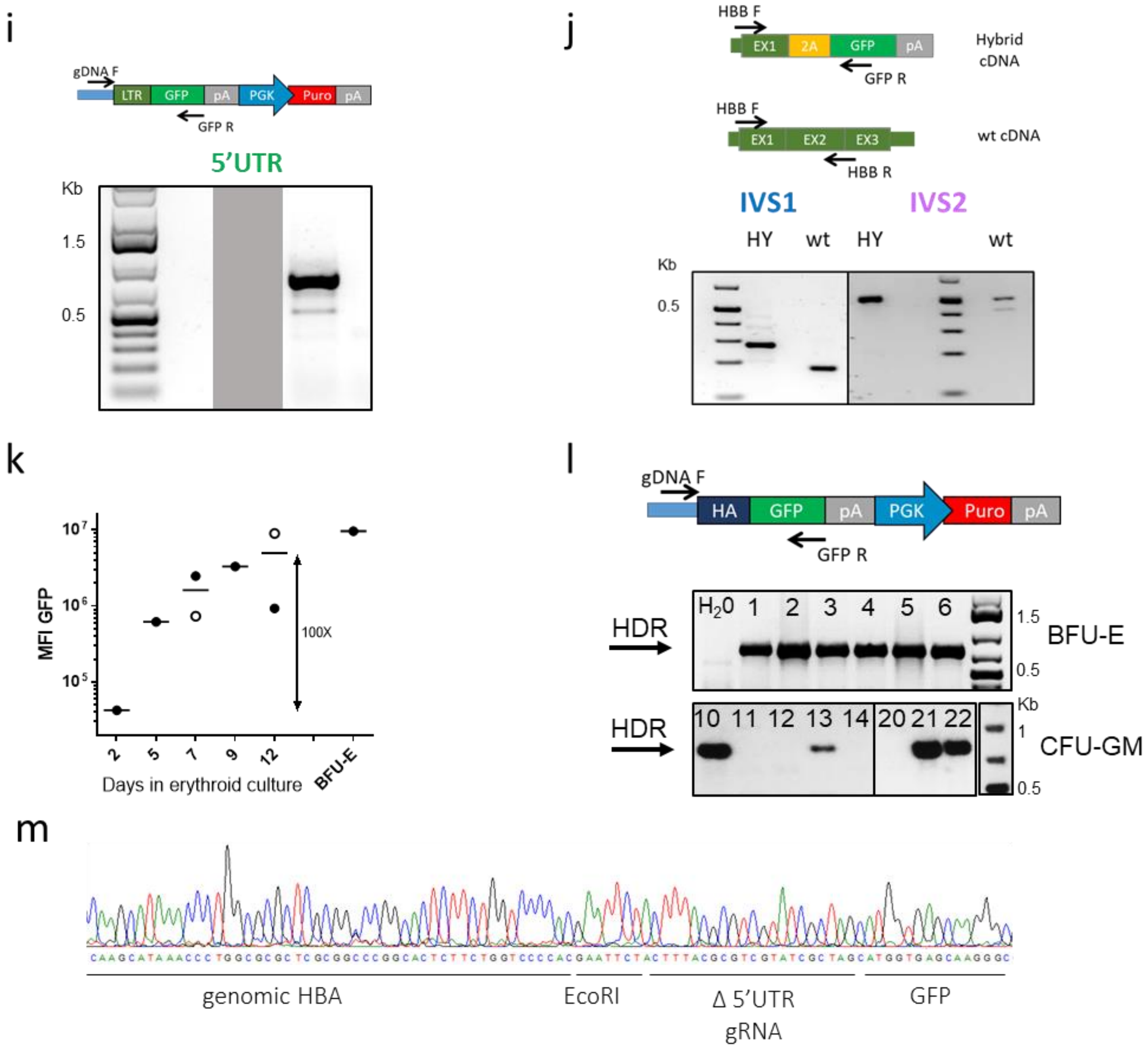

Supplementary Figure 2. (**a**) Proviral form of integrase-defective lentiviral vectors (IDLV) donors used in K562 for KI experiments in 5'UTR (top) and IVS2 (bottom). (**b**) Upregulation of GFP median fluorescent intensity (MFI) in HBA KI K562, upon hemin induction of globin genes. GFP fold increase was calculated in differentiated cells (GYPA+) after normalization of the signal in non-differentiated (GYPA-) cells (n=1). (**c**) PCR analysis of on-target integration in the α-globin locus of K562 KI single-cell clones(24 single-cell clones). A positive control of targeted integration is also shown (+). Schematic representation of the PCR strategy is shown above the graph. (**d**) Detection of the spliced hybrid mRNA (HY), resulting from correct integration and processing of intron traps in IVS1 and IVS2 of HBA(n=1, pooled GFP sorted cells). Schematic representation of the PCR strategy is shown above the graph. WT is the wild type *HBB* mRNA (**e**) Locations of gRNA on *HBB* gene, selected gRNAs are highlighted. (**f**) K562 screening of gRNAs targeting the β-globin locus (5' untranslated region (5'UTR), intron 1 (IVS1) or in intron 2 (IVS2)). Editing efficiency is expressed as percentage of modified *HBB* alleles. Selected gRNAs are highlighted. (n=1, n=2 for

highlighted guides). (**g**) Representative InDel pattern distribution for 5'UTR, IVS1 and IVS2 HBB gRNA as assessed by TIDE. (**h**) Upregulation of GFP median fluorescent intensity (MFI) in *HBB* KI K562, upon hemin induction of globin genes. Same AAVS1 control as in supplementary figure 1b (n=1). (**i**) PCR analysis of on-target integration in the 5'UTR of *HBB* in KI K562 GFP sorted cells (n=1). Schematic representation of the PCR strategy is shown above the graph **(j)** Detection of the spliced hybrid mRNA (HY), resulting from correct integration and processing of intron traps in IVS1 and IVS2 of *HBB* in KI K562 GFP sorted cells (n=1). Schematic representation of the PCR strategy is shown above the graph. WT is the wild type *HBB* mRNA (**k**) GFP median fluorescent intensity (MFI) during differentiation of IVS2 KI HSPCs (bar indicates mean, 2 donors). (**l**) Molecular analysis of single GFP+ BFU-E and random CFU-GM derived from KI HSPCs (n=14). Arrows indicate the amplicon corresponding to the HDR integration of the trap in the 5'UTR of the α-globin locus (n=10). Schematic representation of the PCR strategy is shown above the graph. (**m**) Sanger-sequencing chromatogram of the HDR amplicon (identical for the 10 KI colonies in **l**). Features are indicated below the sequence. Δ5'UTR gRNA indicates the modified sequence in the AAV6 donor to avoid cleavage of the trap after integration.

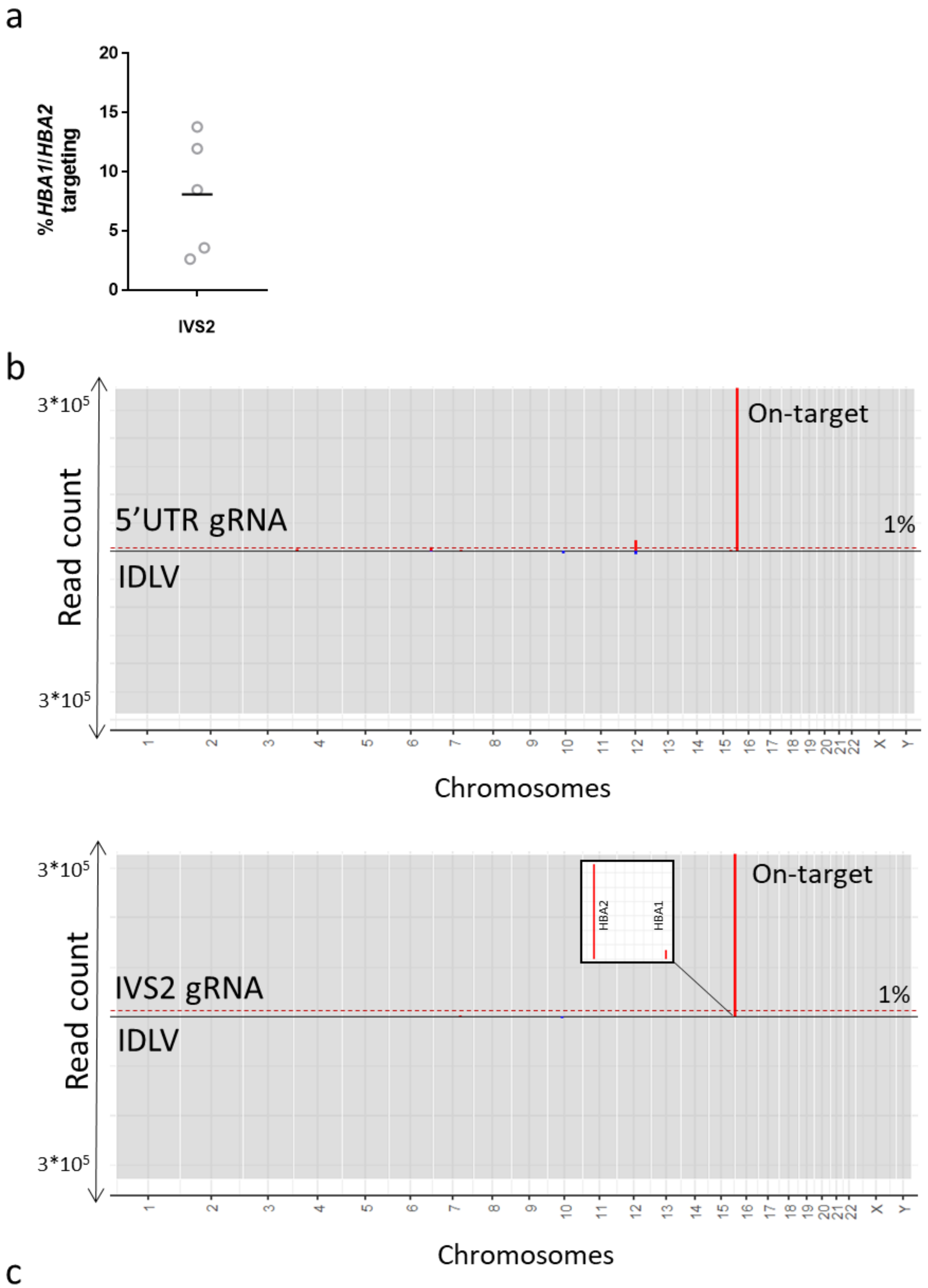

c

Sample		CLIS				Reads					
gRNA	n	total	unique	on-target	gRNA specific*	total	on-target	% on-target	off -target	% off-target	% off-/ on-target
**5'UTR**	3	19	9	2	2**	601,241	556,159	93	-	-	
**IVS2**	2	31	25	1	19$	360,642	328,800	91.2	30,611$$	8.5$$	9.3
**IDLV**	2	6	4	0	-	12,005	-	-	-	-	

*** CLIS present in only one gRNA; ** CLIS present in only 1 replicate out of 3**

$ Only 1 CLIS is present in both replicates (*HBA1*); $$ Data for the *HBA1* main off-target (panel a)

Supplementary Figure 3. (**a**) Percentage of HBA1 editing in K562 after transfection with IVS2 gRNA RNP (n=5). (**b**) Clustered integration sites (CLIS) distribution in different chromosomes in K562 for 5'UTR (top) and IVS2 (bottom) gRNA (n=3 replicates for 5'UTR gRNA, n=2 replicates for IVS2 and IDLV control). Y axis is the number of NGS reads. Red dotted lines indicate 1% of reads. Inset shows results in the genomic interval chr16 220000-227000 (hg19). (**c**) Summary Table of CLIS represented in (**b**). Total = sum of all CLIS in each replicates; unique = number of unique CLIS for each gRNA; gRNA specific: number of unique CLIS for each gRNA after subtraction of shared CLIS observed in different conditions (off-targets).

a

FIX copies	Integration *HBA1*	*HBA2*	*HBA1/ HBA2*
1	19	1	3
2	3		2

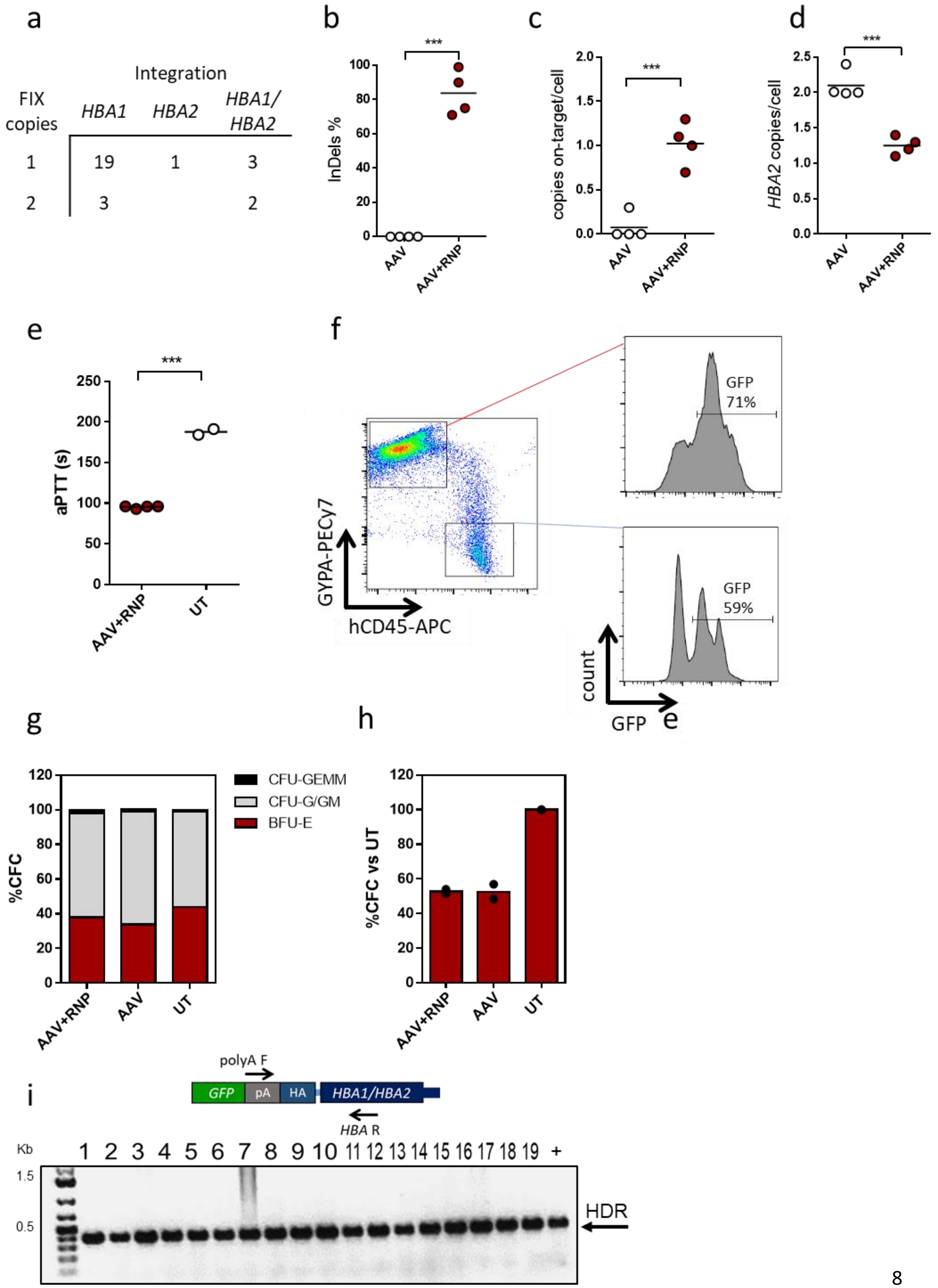

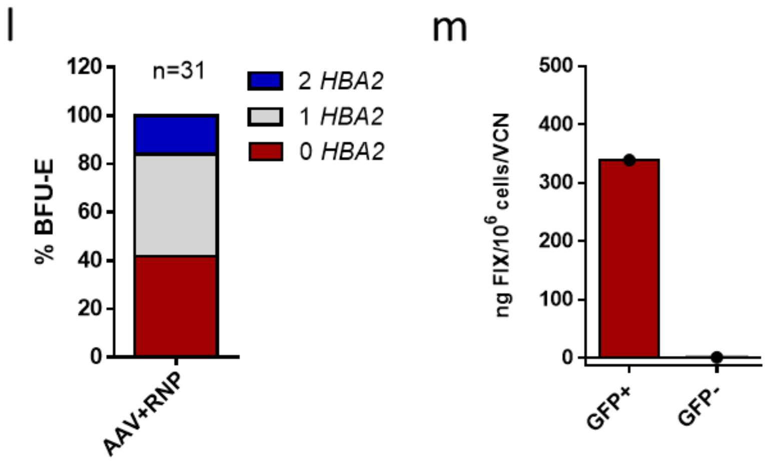

Supplementary Figure 4. (**a**) Characterization of on-target integration of FIX KI HUDEP2 clones shown in Figure 3d. Number of FIX copies integrated and their locations (*HBA1* or *HBA2*) is indicated in the table (n= 28). (**b-d**) Indels quantification (p<0.0001) (**b**), *HBA2* copies (p=0.0004) (**c**) and FIX on-target integration (p=0.0006) (**d**) in edited HSPCs in erythroid liquid culture (n= 4 ; ***: p <0.001, two-tailed Student's t test). (**e**) Raw clotting times (seconds, s) from supernatants of KI (AAV+RNP) or untreated (UT) HSPC (n= 4 AAV+RNP, n=2 UT; ***: p<0.0001, two-tailed Student's t test). (**f**) Representative cytometry dot plots showing GFP expression in red (GYPA+) and white (CD45+) CFC (from 3 biological replicates). (**g**) Colony formation unit (CFU) frequency in KI HSPC. CFU-GEMM, granulocyte, erythroid, macrophage, megakaryocyte; BFU-E, burst-forming unit-erythroid; CFU-G/GM, granulocyte-macrophage. Bars represent mean ±SD (n= 2 AAV+RNP and UT, n=3 AAV). (**h**) CFC number expressed as percentage of untreated control (UT). Bars represent mean ±SD (n=2). (**i**) PCR analysis of single GFP+ BFU-E derived from FIX KI HSPCs. Arrow indicates the amplicon size compatible with HDR mediated integration of the trap in the 5'UTR of *HBA* (n=19). A positive control colony is also shown (+). Schematic representation of the PCR strategy is shown above the graph. (**l**) Quantification of *HBA2* copies in single BFU-E of FIX KI HSPCs. (**m**) FIX expression in supernatants of GFP sorted BFU-E of KI-HSPCs (n=1, pooled from 2 replicates).

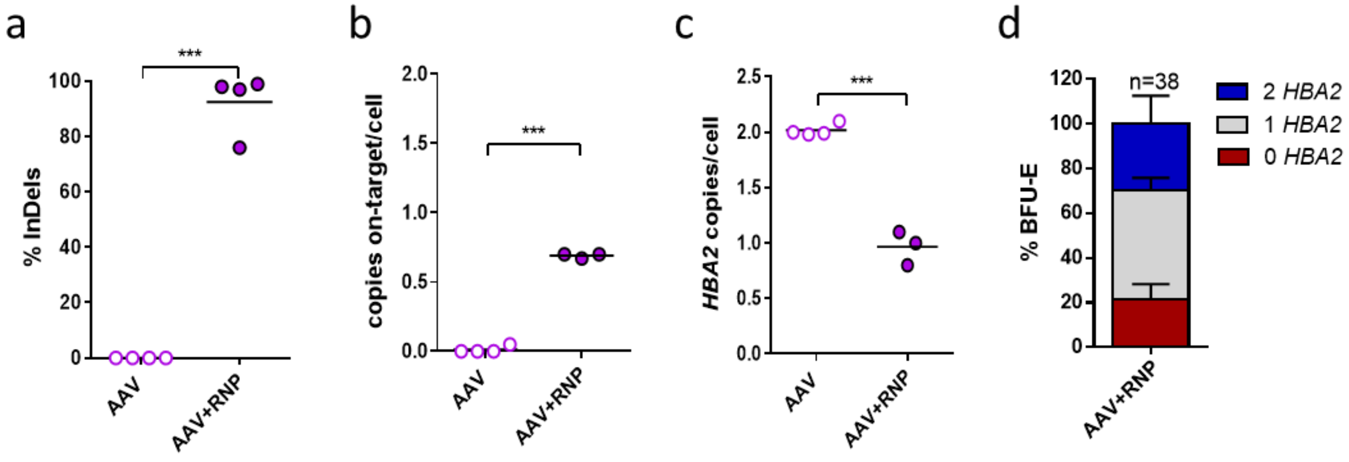

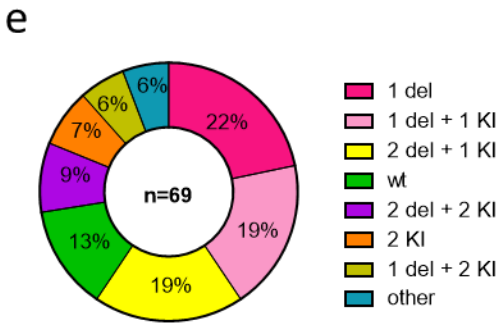

f

On-target integration	*HBA2* copies 2	1	0
0	9	15	2
1	2	13	13
2	5	4	6

Supplementary Figure 5. Indels quantification (**a**), LAL on-target integration (**b**) and *HBA2* copies (**c**) in edited HSPCs in erythroid liquid culture(n=3-4; ***: p <0.001, Student's t test). (**d**) Quantification of *HBA2* copies in single BFU-E of LAL KI HSPCs. Bars represent mean ±SD (colonies derived from 2 donors). (**e**) Genotypes of BFU-E derived from FIX and LAL KI HSPCs. ("wt", unmodified colonies; "KI", on-target integration; "del", *HBA2* deletion). Percentages are indicated (n=69, 3 donors). (**f**) Colony genotypes shown in (**e**).

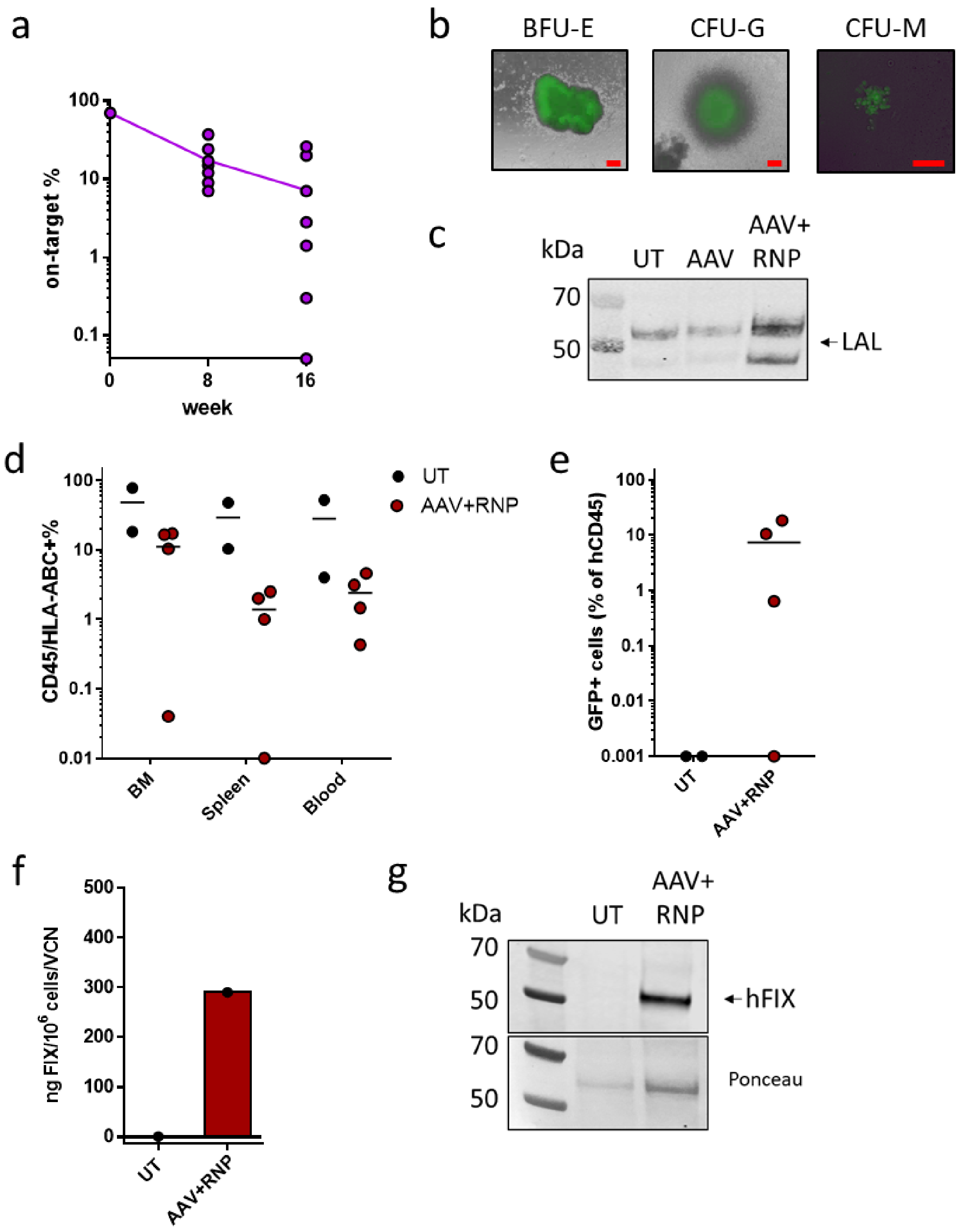
a
100
10
1
0.1
on-target %
0
8
16
week
b
BFU-E
CFU-G
CFU-M
c
kDa
UT
AAV
AAV+
RNP
70
50
LAL
d
100
10
1
0.1
0.01
CD45/HLA-ABC+%
BM
Spleen
Blood
UT
AAV+RNP
e
100
10
1
0.1
0.01
0.001
GFP+ cells (% of hCD45)
UT
AAV+RNP
f
500
400
300
200
100
0
ng FIX/10^6 cells/VCN
UT
AAV+RNP
g
kDa
UT
AAV+
RNP
70
50
hFIX
70
50
Ponceau

Supplementary Figure 6. (**a**) Percentage of on-target integration in mouse peripheral blood by ddPCR. (**b**) Representative overlay images (bright field and GFP channel) of KI HSPC progenitors recovered from transplanted mice. BFU-E, burst-forming unit-erythroid; CFU-G, granulocyte; CFU-M, monocyte. Scale bars in red indicate 200 µm. (**c**) Western blot of LAL in CD34-derived BFU-E from mice that received untreated (UT) and KI-HSPCs (AAV+RNP). Anti-LAL tag antibody was used. (**d**) Percentage of human CD45+/HLA-ABC+ cells in hematopoietic organs of mice at week 16. BM = bone marrow (n= 4 AAV+RNP, n=2 UT). (**e**) Edited cells in bone marrow of transplanted mice. GFP is expressed as percentage of CD45+ cells, mean is shown (n= 4 AAV+RNP, n=2 UT). (**f**) Human FIX expression in supernatants of CD34-derived BFU-E from mice that engrafted with untreated (UT) or KI-HSPCs (AAV+RNP) (n= 1, pooled from 2 mice). (**g**) Western blot on CD34-derived BFU-E from mice engrafted with untreated (UT) or KI HSPCs (AAV+RNP). Ponceau staining is also shown (n= 1, pooled from 2 mice).

Supplementary table 1: List of gRNAs tested

**HBA gRNA name**	**Protospacer with <u>PAM</u>**
HBA 10	GGGTTTATGCTTGGGGCGCG**<u>GGG</u>**
HBA 12	GACTCAGAGAGAACCCACCA**<u>TGG</u>**
HBA 14	TGGGTTCTCTCTGAGTCTGT**<u>GGG</u>**
HBA 19	GCGCGGGGGCACGCCCGGCC**<u>GGG</u>**
HBA 15 (5'UTR)	GGGTTCTCTCTGAGTCTGTG**<u>GGG</u>**
HBA 16 (KO)	GTCGGCAGGAGACAGCACCA**<u>TGG</u>**
HBA 17	GCAGGAGACAGCACCATGGT**<u>GGG</u>**
HBA 20	CATAAACCCTGGCGCGCTCG**<u>CGG</u>**
HBA INT1 73b (IVS1)	CAGGCCACCCTCAACCGTCC**<u>TGG</u>**
HBA INT1 72	TCCGGGGCCAGGACGGTTGA**<u>GGG</u>**
HBA INT1 73	GTCCGGGGCCAGGACGGTTG**<u>AGG</u>**
HBA INT2 13REV	CCCTCGACCCAGATCGCTCC**<u>CGG</u>**
HBA INT2 63	GAAGAGGGTCAGTGCGGCCC**<u>AGG</u>**
HBA INT2 74 (IVS2)	GCGTGATCCTCTGCCCTGAG**<u>AGG</u>**
**HBB gRNA name**	**Full Sequence with <u>PAM</u>**
HBB 37	GGTTGGCCAATCTACTCCC**<u>AGG</u>**
HBB 49	GGGTTGGCCAATCTACTCCC**<u>AGG</u>**
HBB 53	GGAGTAGATTGGCCAACCCT**<u>AGG</u>**
HBB 54	GATTGGCCAACCCTAGGGTG**<u>TGG</u>**
HBB 77 (5'UTR)	GAGTAGATTGGCCAACCCTA**<u>GGG</u>**
HBB INT 1 36 (IVS1)	TGGTATCAAGGTTACAAGAC**<u>AGG</u>**
HBB INT1 36REV	TCCACATGCCCAGTTTCTAT**<u>TGG</u>**
HBB INT1 47FOR	TTAAGGAGACCAATAGAAAC**<u>TGG</u>**
HBB INT1 48FOR	TAAGGAGACCAATAGAAACT**<u>GGG</u>**
HBB INT2 340FOR	CTGCCTAGTACATTACTATT**<u>TGG</u>**
HBB INT2 797REV	ATTAGCAAAAGGGCCTAGCT**<u>TGG</u>**
HBB INT2 20 (IVS2)	GTTAAGTTCATGTCATAGGA**<u>AGG</u>**
HBB INT2 39	GACGAATGATTGCATCAGTG**<u>TGG</u>**
**Control**	**Full Sequence with <u>PAM</u>**
AAVS1	GTCCCCTCCACCCCACAGTG**<u>GGG</u>**

Supplementary table 2: 5'UTR gRNA off-targets

Off Target	Sequence	mismatch	deletion	chr	start	end	strand	type	gene	Indel % HBA15 *	Indel % AAVS1*
1	GGGTTTTCCTGAGTCTGTGTGG	1	Del 12	chr6	167624159	167624180	-	intergenic	TCP10L2-UNC93A	0	1
2	GGGTTCCCTCTGATCTGTGGGG	1	Del 7	chr7	57350881	57350902	-	intergenic	LOC105375297-LOC100653233	1	1
3	GGGTTCCTCTGAGGCTGTGAGG	1	Del 14	chr21	35358171	35358192	+	intergenic	LOC101928126-SLC5A3	0	0
4	GGGTTTCTCTGAGTCAGTGTGG	1	Del 15	chr14	38170058	38170079	-	intron	TTC6	0	0
5	GGGTTTCTCTGAGTCTCTGGGG	1	Del 15	chr17	77153181	77153202	-	intron	RBFOX3	0	1
6	GGGTGCTCTCTGAGTCTGGTGGGG	1	Ins 3	chr9	93549548	93549571	-	intergenic	DIRAS2-SYK	0	ND
7	GGGTTCTCTCTGAGCCTGGAGG	1	Del 2	chr2	231805880	231805901	-	intergenic	GPR55-SPATA3-AS1	3	4
8	CGGTTCTCTCTGAGTCTATGAAG	2	0	chr6	169440547	169440569	-	intergenic	RP3-495K2.2-RP3-495K2.1	1	1
9	GGGTTCCCTCTGAGGCTGTGAGG	2	0	chr9	77688205	77688227	-	intron	NMRK1	1	1
10	GGGTTCTCTCTCAGTCTGTGTGT	2	0	chr6	24657192	24657214	-	intron	TDP2	0	0
11	TGATTCTTTCTGAGTCTGTGAGG	3	0	chr2	229878873	229878895	-	intergenic	AC007677.2-PID1	0	0
12	TTGTGCTCTCTGAGTCTGTGTGG	3	0	chr16	58978340	58978362	+	intergenic	RP11-700H13.1-RP11-410D17.2	0	0
13	TGCTTCTCTCTGAGTCTGGGCGA	3	0	chr8	134399964	134399986	-	intergenic	CTC-458A3.1-CTC-369M3.1	1	1
14	GGATTCTGTCTGTGTCTGTGAGA	3	0	chr11	66956537	66956559	-	intergenic	KDM2A-AP001885.1	1	1
15	GGGTTCTTTCTGAGGCTGGGAGG	3	0	chr7	138374829	138374851	-	intergenic	AC020983.5-SVOPL	0	0
16	GGGTTCTCTCTGAGGCTTTCAGG	3	0	chr16	31075849	31075871	-	intron	ZNF668	2	0
17	TGTTTCTATCTGAGTCTGTGGGA	3	0	chr16	54632788	54632810	-	intergenic	AC079412.1-RP11-1136G4.1	0	0
18	TGGTTCTCTCTGAGGCTCTGAGG	3	0	chr8	145594499	145594521	+	intergenic	SLC52A2-ADCK5	ND	ND
19	GGGTTCGTTCTGAGGCTGTGAGG	3	0	chr11	2577287	2577309	+	intron	KCNQ1	1	1
20	GGGTTTGCTCTGAGGCTGTGGGG	3	0	chr4	1392471	1392493	+	intergenic	CRIPAK-NKX1-1	0	1
21	AGATGCTGTCTGAGTCTGTGGGG	4	0	chr12	4038222	4038244	+	intergenic	RP11-664D1.1-RP11-320N7.2	1	0
22	ATGACCTCTCTGAGTCTGTGTGG	4	0	chr3	127328104	127328126	+	intron	MCM2	1	2
23	TGTTTCTGTGTGAGTCTGTGTGG	4	0	chr19	55433833	55433855	-	intergenic	NCR1-NLRP7	ND	ND
24	GATATCTCTGTGAGTCTGTGAGG	4	0	chr3	70751897	70751919	+	intergenic	RP11-231I13.2-COX6CP6	1	0
25	TGGTCCTCTCAGAGTCTGTAAGG	4	0	chr20	3236843	3236865	+	exon	C20orf194	1	2
26	TGATTCTTTCTGAGTATGTGGGG	4	0	chr10	37317185	37317207	+	intergenic	ARL6IP1P2-ANKRD30A	1	0
27	TGATTCTTTCTGAGTATGTGGGG	4	0	chr11	80331104	80331126	-	intergenic	RNU6-544P-ARL6IP1P3	1	0
28	GGGTTTTCTTAGAATCTGTGAGG	4	0	chr11	48672787	48672809	+	intergenic	OR4A44P-RP11-56P9.5	not present in K562	
29	GGGTCTTCCCTCAGTCTGTGTGG	4	0	chr5	154003375	154003397	-	intergenic	MIR3141-MIR1303	0	0

*With a threshold limit of ~2% (TIDE[22])

Supplementary table 3: primer list

PCR		
**Gene/amplicon**	**name**	**sequence**
HBA 1/2 – IVS1	3820 F	TATCGCCAGAGGGAAAGGGA
	4870 R	CTTGAAGTTGACCGGGTCCA
HBA1/2 sequencing	894 R	TAGGTCTTGGTGGTGGGGAA
HBA2	611 F	GCACTCTTCTGGTCCCCAC
	1512R	GCAGAGAGGTCCTTGGTCTG
HBA1	HBA EX1F	CCGACAAGACCAACGTCAA
	5614 R	CTCTAGGGTCCAGCGTTTTTCC
AAVS1	MA359	CAGCTCAGGTTCTGGGAGAG
	MA360	CTTGTAGGCCTGCATCATCA
HBB 5UTR	HBB9 F	CTGTCTCCACATGCCCAGTT
	HBB9 R	GGAGACGCAGGAAGAGATCC
HBB IVS2	HBB IVS2 F	TTGGACAGCAAGAAAGCGAG
	HBB IVS2 R	GTGAGTCTATGGGACGCTTGA
HBA EX1-2 cDNA	HBA EX1F	CCGACAAGACCAACGTCAA
	4870 R	CTTGAAGTTGACCGGGTCCA
HBA-GFP IVS1 hybrid	HBA EX1F	CCGACAAGACCAACGTCAA
	PPT_EGFP_R	GAACTTCAGGGTCAGCTTGC
HBA EX2-3 cDNA	875 F	GAACTTCAGGGTCAGCTTGC
	HBA EX3 R	CTCACAGAAGCCAGGAACTT
HBA-GFP IVS2 hybrid	875 F	TTCCCCACCACCAAGACCTA
	PPT_EGFP_R	GAACTTCAGGGTCAGCTTGC
On-target 5'junct (5'UTR - IVS1 HBA)	3820 F	TATCGCCAGAGGGAAAGGGA
	PPT_EGFP_R	GAACTTCAGGGTCAGCTTGC
On-target 5'junct (IVS2 HBA)	HBA EX1F	CCGACAAGACCAACGTCAA
	PPT_EGFP_R	GAACTTCAGGGTCAGCTTGC
HBA2 INT	mini PolyA 1F	TGGACAAACCACAACTAGAATGC
	1512R	GCAGAGAGGTCCTTGGTCTG
HBA1 INT	mini PolyA 1F	TGGACAAACCACAACTAGAATGC
	5614 R	CTCTAGGGTCCAGCGTTTTTCC
OFF TARGET 1	OFF-T 1COSF	CTTTAGACACCCAGTGGGAAG
	OFF-T 1COSF	AGCCAGATGCCATAGAGTTTAC
OFF TARGET 2	OFF-T 2COSF	CTGCAACTGAACCCGAGTAATA
	OFF-T 2COSR	GGGAGTAGGCAGCATGATTT
OFF TARGET 3	OFF-T 4COSF	ACCCTCCTCACACCATGTA
	OFF-T 4COSR	ACCTCTGAATGTGGCTTTATGT
OFF TARGET 4	OFF-T 5COSF	CCAAGGCAGGCAGATTACTT
	OFF-T 5COSR	GACAACAGTCTACAGGCATCTC
OFF TARGET 5	OFF-T 6COSF	TTTCATCCCTGGCAAGCTAC
	OFF-T 6COSR	TTTGTGGTCAGTGTCTGTGG
OFF TARGET 6	OFF-T 7COSF	ACCTCTAGGATGAGAGGTAAA
	OFF-T 7COSR	CTATTGGATTAGATAGCATGTTGTG
OFF TARGET 7	OFF-T 8COSF	CCTGCAGGGAGGAATTAAGAAG

	OFF-T 8COSR	CCCTGGGTTCAGGTTCAAAT
OFF TARGET 8	OFF-T HBA15 1F	TAAAGTCCTAATGCCCAGTTCTC
	OFF-T HBA15 1R	CTGCCCAGGTTCAAACAATTC
OFF TARGET 9	OFF-T 9COSF	TCAGCCTTATCACCCATCATC
	OFF-T 9COSR	GCTCTGCAGAACCCATCTAT
OFF TARGET 10	OFF-T HBA15 2F	GAACTGGGTCATAGCTGGGG
	OFF-T HBA15 2R	CATCCTGTCTCCATCGCAGG
OFF TARGET 11	OFF-T 1MIT47 F	CGCAGACACAGACACTCAAA
	OFF-T 1MIT47 R	CTCCTTCACAGAAACCCATCC
OFF TARGET 12	OFF-T 2MIT45F	TATGTGGCTTCCCTTGGTTC
	OFF-T 2MIT45R	TGACCTTTGCTGTGCTTAGT
OFF TARGET 13	OFF-T HBA15 3F	TGTGTAGATGGGTTCTCTACCT
	OFF-T HBA15 3R	CTACCCTTGGAGAACTGTATGC
OFF TARGET 14	OFF-T HBA15 4F	TGCAGTGACCTGAGATTGTG
	OFF-T HBA15 4R	CCCATTGTGGTTGGAGAAGATA
OFF TARGET 15	OFF-T HBA15 5F	TCACAAGGTCAGCAGTTTGA
	OFF-T HBA15 5R	TGCGTGGACACGTGTATTAG
OFF TARGET 16	OFF-T 6bMIT259F	TCACGATTCCTCAAGCTACAC
	OFF-T HBA15 6R	CACGTCTGGGTACACTCTTTATC
OFF TARGET 17	OFF-T HBA15 7F	AAGGTGAAGGAGGAGCAAAG
	OFF-T HBA15 7R	GTGAGGAGGCTGAAACGATAG
OFF TARGET 18	OFF-T HBA15 8F	CTGGCTAACACGGTGAAACT
	OFF-T HBA15 8R	CAGGAGAAGAGAGAGGGAGATT
OFF TARGET 19	OFF-T HBA15 9F	CCTCCCATATGCCCACATTT
	OFF-T HBA15 9R	ATCAGCAGCCAGGTTTGTAG
OFF TARGET 20	OFF-T HBA15 10F	GGATGTGAGTAGATCAGGTTGG
	OFF-T HBA15 10R	TGGCAATGGTGTGCTTCT
OFF TARGET 21	OFF-T 4MIT329F	GCATGTGTGTGCATGTGTATG
	OFF-T 4MIT329R	CTGTGGCCCTAATCCTTTGTAG
OFF TARGET 22	OFF-T 5MIT327F	CAGGATGCTGTGAGAGGATATG
	OFF-T 5MIT327R	GATCTTGAGCCAGGAGTTTGA
OFF TARGET 23	OFF-T 6MIT259F	AGATCACGAGGTCAGGAGAT
	OFF-T 6MIT259R	CTTCTTGGGCCATTTGCTATTT
OFF TARGET 24	OFF-T 7MIT206F	CCCTTTCCACTTTCCTGGTAAC
	OFF-T 7MIT206R	GCAAGCGAAGGAGGTCATTTA
OFF TARGET 25	OFF-T 1CFD355F	CCAGTCCTTGATGCTGTCTT
	OFF-T 1CFD355R	TGTTCCTCTCTGTCTCTCTCTC
OFF TARGET 26	OFF-T 3CFD353F	CCGGTTGCTATCTGTGGAAATA
	OFF-T 3CFD353R	CTAGGAGAGACAATCACCATGC
OFF TARGET 27	OFF-T 4CFD354F	CACCATGATTTCGGGAGTTTG
	OFF-T 4CFD354R	CAGACACTGCTCTACACTATCC

OFF TARGET 28	OFF-T 5CFD352F	GTTAGGGATGTGGGCATTCA
	OFF-T 5CFD352R	GGTTTCTCAGAGAGCTTCCTTC
OFF TARGET 29	OFF-T 6CFD351F	GTGGTGGTACTCTCCTGTAGT
	OFF-T 6CFD351R	CCTGCTGTGTTCCTTCCTTT
**ddPCR**		
On-target 3'junction	3'HBA INT F	TGGACAAACCACAACTAGAATGC
	3'HBA INT 3 R	AAGTGCGGGAAGTAGGTCTT
	3'HBA INT PRB	56-FAM/CTGTCTCCTGCCGACAAGACCAAC
HBA2	HBA2 3' F	GCCCTTCCTGGTCTTTGAATA
	HBA2 3' R	ACCTCCATTGTTGGCACAT
	HBA2 3' PRB	56-FAM/TGTGTGTGCCTGGGTTCTCTCTAT
ALB	ALB F	GCTGTCATCTCTTGTGGGCTGT
	ALB R	ACTCATGGGAGCTGCTGGTTC
	ALB P	CCTGTCATGCCCACACAAATCTCTCC
**qPCR**		
Human FIX	hFIX 1F	AAGCGGTACAACTCAGGCAA
	hFIX 1R	CTTCCAGAACTCGGTGGTCC
HA-tag	HATAG 1F	TGATTACGCCGGCACAG
	HATAG 1R	ATCAGGCACATCATAAGGGTATC
GAPDH	GAPDH F	CTTCATTGACCTCAACTACATGGTTT
	GAPDH R	TGGGATTTCCATTGATGACAAG

F=forward; R=reverse, PRB=probe.

Supplementary table 4: Antibodies for flow cytometry

Name	Fluorochrome	Clone	Company	Catalog Number	Dilution
human Fc Receptor binding inhibitor polyclonal antibody			eBioscience	16-9161-73	1:50
anti-human Fetal Hemoglobin	APC		Life Technologies	MHF05	Per manufacturer's instructions
anti-human Hemoglobin β	PE	37-8	Santa Cruz Biotechnology	sc-21757	1:50
anti-human HLA-ABC	PE	G46-2.6	BD Bioscience	555553	Per manufacturer's instructions
anti-human CD36	FITC	C836	BD Bioscience	555454	Per manufacturer's instructions
anti-human CD235a (GYPA)	PE-Cy7	GA-R2	BD Bioscience	563666	Per manufacturer's instructions
anti-human CD71	APC	M-A712	BD Bioscience	551374	Per manufacturer's instructions
anti-human CD45	APC	HI30	BD Bioscience	555485	Per manufacturer's instructions
anti-human CD45	PB	J.33	Beckman Coulter	A74765	Per manufacturer's instructions
anti-human CD33	BV421	P67.6	BD Bioscience	744761	1:100
anti-human CD19	BV605	HIB19	BD Bioscience	740394	1:100
anti-human CD3	BB700	SK7	BD Bioscience	566575	1:10
anti-human CD34	PE	AC136	Miltenyi Biotec	130-113-179	1:50
anti-human CD38	APC	REA671	Miltenyi Biotec	130-110-244	1:25
IgG1, κ isotype control	APC		Life Technologies	MG105	Per manufacturer's instructions
IgG1, κ isotype control	APC		BD Bioscience	550854	Per manufacturer's instructions
IgG1, κ isotype control	BV421		BD Bioscience	562438	1:100
IgG1, κ isotype control	BV605	X40	BD Bioscience	562652	1:100
IgG1, κ isotype control	BB700	X40	BD Bioscience	566404	1:100

IgG2a, κ isotype control	APC	G155-178	BD Bioscience	551414	Per manufacturer's instructions
IgG2a, κ isotype control	PE		BD Bioscience	555574	Per manufacturer's instructions
IgG2a, κ isotype control	PE	S43.10	Miltenyi Biotec	130-113-272	1:50
IgG2b, κ isotype control	PE-Cy7		BD Bioscience	560542	1:100

All antibodies were mouse monoclonal antibodies, unless differently specified.

Supplementary table 5: Antibodies for western Blot

Target	host	Fluorochrome	Clone	Company	Dilution
anti-human LAL	Mouse	-	9G7F12, 7G6D7	Thermo-Fisher	1:1000
HA-TAG	Mouse	-	16B12	Eurogentec	1:1000
anti-human α-globin	Goat	-	D-16	Santa Cruz Biotechnology	1:200
anti-human FIX	Rabbit	-	Polyclonal	Invitrogen	1:500
anti-human β-tubulin	Rabbit	-	Polyclonal	Li-Cor Biosciences	1:1000
Anti-mouse IgG (H+L)	Goat	800CW	Polyclonal	Li-Cor Biosciences	1:15000
Anti-mouse IgG (H+L)	Donkey	680LT	Polyclonal	Li-Cor Biosciences	1:15000
Anti-rabbit IgG (H+L)	Goat	680LT	Polyclonal	Li-Cor Biosciences	1:15000
Anti-goat IgG (H+L)	Donkey	800CW	Polyclonal	Li-Cor Biosciences	1:15000

Supplementary Methods

Intron trap design: sequence details

Partial nucleotide sequence of IVS2 trap. Synthetic intron (IVS, gray), splice acceptor (SA), a frame adaptor (light blue), self-cleaving peptide from porcine teschovirus-1 (P2A, orange), and the 5′ end of GFP (green) are shown. Single-letter amino acid code is indicated below the sequence. IVS1 trap was similar to IVS2 trap with the exclusion of the 8 nucleotides underlined in the frame adaptor sequence.

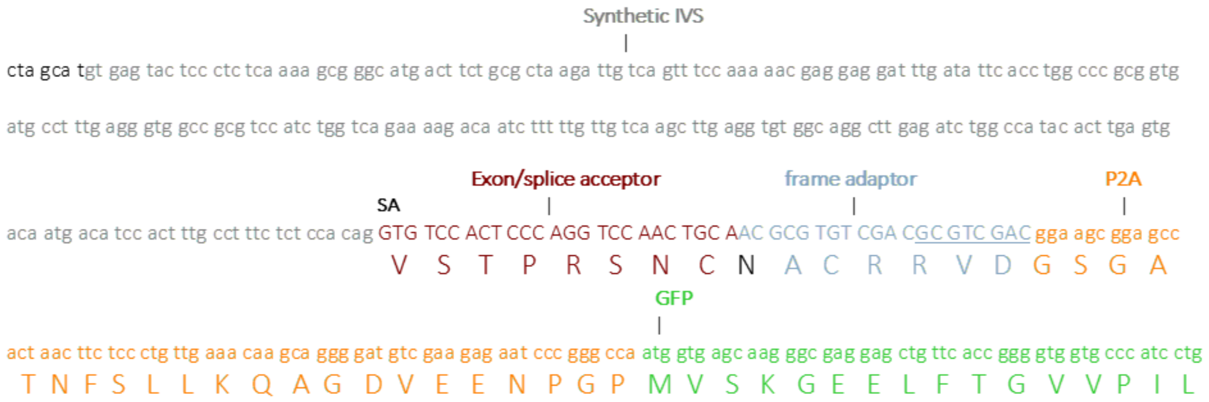

Validation of digital droplet PCR (ddPCR) for on target integration: correlation of flow cytometry and molecular data

Correlation of GFP expressing cells assessed by flow cytometry and ddPCR for on-target integration. Linear regression of data with 95% confidence band is shown.

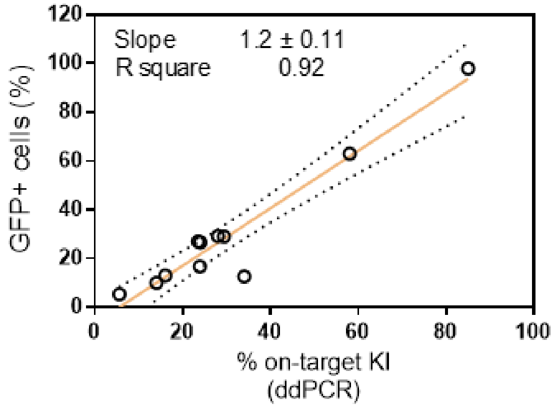

Gating strategy for immunophenotyping of erythroid markers

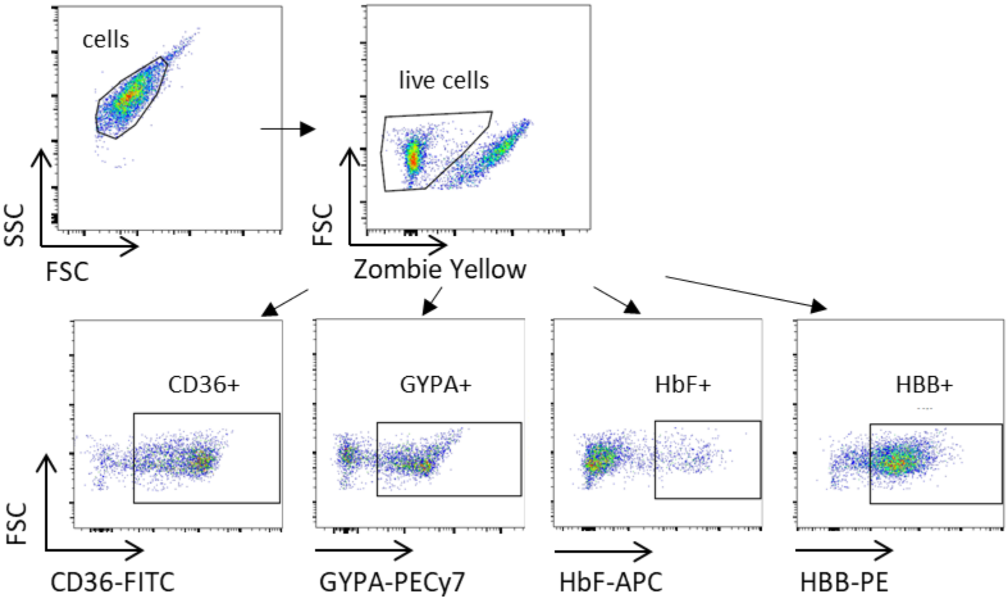

Gating scheme used to analyze erythroid markers on differentited erythroblasts. Representative plots for quantification of CD36 (CD36+), glycophorin A (GYPA+) and fetal hemoglobin (HbF+) and β-globin (HBB+) expressing cells.

Gating strategy for immunophenotyping of human engrafted cells in NSG mice
Gating scheme used to analyze human cell engraftment and cell lineages after mouse transplantation. Representative plots for quantification human hematopoietic (CD45+/HLA-ABC+), human B (CD19+), human myeloid (CD33+) and human T (CD3+) cells.

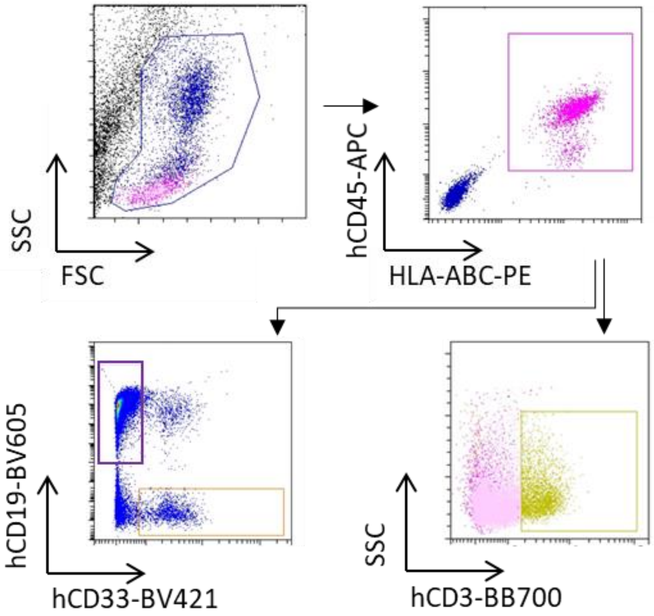

Supplement: Supplementary file 1 — Supplementary Information [file 41467_2020_17552_MOESM1_ESM.pdf]
