# Supplementary material for: Ex vivo editing of human hematopoietic stem cells for erythroid expression of therapeutic proteins
Source: Nat Commun. 2020 Jul 29;11:3778. doi: 10.1038/s41467-020-17552-3 (PMC7391635; doi:10.1038/s41467-020-17552-3)
Supplement: Supplementary file 2 — Reporting Summary [file 41467_2020_17552_MOESM2_ESM.pdf]

nature research

Corresponding author(s): Mario Amendola

Last updated by author(s): Apr 11, 2020

Reporting Summary

Nature Research wishes to improve the reproducibility of the work that we publish. This form provides structure for consistency and transparency in reporting. For further information on Nature Research policies, see Authors & Referees and the Editorial Policy Checklist .

Statistics

For all statistical analyses, confirm that the following items are present in the figure legend, table legend, main text, or Methods section.

n/a	Confirmed	
☐	☒	The exact sample size (*n*) for each experimental group/condition, given as a discrete number and unit of measurement
☐	☒	A statement on whether measurements were taken from distinct samples or whether the same sample was measured repeatedly
☐	☒	The statistical test(s) used AND whether they are one- or two-sided<br>*Only common tests should be described solely by name; describe more complex techniques in the Methods section.*
☐	☒	A description of all covariates tested
☐	☒	A description of any assumptions or corrections, such as tests of normality and adjustment for multiple comparisons
☐	☒	A full description of the statistical parameters including central tendency (e.g. means) or other basic estimates (e.g. regression coefficient) AND variation (e.g. standard deviation) or associated estimates of uncertainty (e.g. confidence intervals)
☐	☒	For null hypothesis testing, the test statistic (e.g. *F*, *t*, *r*) with confidence intervals, effect sizes, degrees of freedom and *P* value noted<br>*Give P values as exact values whenever suitable.*
☒	☐	For Bayesian analysis, information on the choice of priors and Markov chain Monte Carlo settings
☒	☐	For hierarchical and complex designs, identification of the appropriate level for tests and full reporting of outcomes
☒	☐	Estimates of effect sizes (e.g. Cohen's *d*, Pearson's *r*), indicating how they were calculated

Our web collection on statistics for biologists contains articles on many of the points above.

Software and code

Policy information about availability of computer code

Data collection	QuantaSoft software,Illumina MiSeq, ABI Prism 7900HT Sequence detection system, Light Cycler480, SPARK TECAN Reader, Odyssey imager, EnSpire software, CytExpert, SP6800 software
Data analysis	Prism 7 GraphPad Software, Microsoft Excel, FlowJo, CytExpert, QuantaSoft, COSMID, TIDE, CRISPOR, ImageJ, Magellan Software, ImageStudio Lite, Vector NTI, Serial Cloner

For manuscripts utilizing custom algorithms or software that are central to the research but not yet described in published literature, software must be made available to editors/reviewers. We strongly encourage code deposition in a community repository (e.g. GitHub). See the Nature Research guidelines for submitting code & software for further information.

Data

Policy information about availability of data

All manuscripts must include a data availability statement. This statement should provide the following information, where applicable:

- Accession codes, unique identifiers, or web links for publicly available datasets
- A list of figures that have associated raw data
- A description of any restrictions on data availability

The authors declare that data supporting the findings of this study are available within the paper and its supplementary information files or from the corresponding author on reasonable request.
Raw and processed data of Off-target analyisis are available from the NCBI Gene Expression Omnibus (GEO) (https://www.ncbi.nlm.nih.gov/geo/query/acc.cgi?acc=GSE133861) under accession number GSE133861.

Field-specific reporting

Please select the one below that is the best fit for your research. If you are not sure, read the appropriate sections before making your selection.

- [x] Life sciences
- [ ] Behavioural & social sciences
- [ ] Ecological, evolutionary & environmental sciences

For a reference copy of the document with all sections, see nature.com/documents/nr-reporting-summary-flat.pdf

Life sciences study design

All studies must disclose on these points even when the disclosure is negative.

Sample size	The minimum sample size was determined by the amount of primary HSPCs and mice needed to parse out statistical significance.<br>Statistical analyses used in this study are: one-way or two-way Anova, Tukey's test, Dunnet's test, two-tailed Mann Whitney test, Holm-Sidak test, two-tailed Student's t test.
Data exclusions	No data were excluded from the analysis.
Replication	Experimental and biological replicates were performed for every experiment. Every measurement was performed in duplicate or triplicate (technical replicates).<br>All attempts at replicating the experiments were successful with the exception of in vivo reconstitution of immune system with edited CD34+ HSPCs. This was due to due to the overall low levels of human engraftment in transplanted mice.
Randomization	Mice were randomly assigned to different treatment groups. We used female NSG mice as recipient as CD34+ HSPCs were derived from both male and female healthy donors.<br>The type of WD pathogenic mutation used in the study was random and based on the availability of such samples.
Blinding	Microscopy images were taken by blinded observers. Image quantification was performed by publicly available macros and softwares. For the other experiments, blinding was not relevant.

Reporting for specific materials, systems and methods

We require information from authors about some types of materials, experimental systems and methods used in many studies. Here, indicate whether each material, system or method listed is relevant to your study. If you are not sure if a list item applies to your research, read the appropriate section before selecting a response.

Materials & experimental systems

n/a	Involved in the study	
[ ]	[x]	Antibodies
[ ]	[x]	Eukaryotic cell lines
[x]	[ ]	Palaeontology
[ ]	[x]	Animals and other organisms
[x]	[ ]	Human research participants
[x]	[ ]	Clinical data

Methods

n/a	Involved in the study	
[x]	[ ]	ChIP-seq
[ ]	[x]	Flow cytometry
[x]	[ ]	MRI-based neuroimaging

Antibodies

Antibodies used	Antibodies list is included in supplementary data. Add dilution
Validation	Antibodies were used per manufacturer's instructions. When not available, an antibody titration was performed using antigen positive and antigen negative cells as well as isotype controls from the same manufacturer.<br>Add link to manufacturer

Eukaryotic cell lines

Policy information about cell lines

Cell line source(s)	K562 (ATCC® CCL-243); HUDEP-2 (Kurita, Plos one 2013)
Authentication	K562 (ATCC® CCL-243) and HUDEP-2 were karyoptyped, presence of multiple erythorid markers were analyzed by flow cytometry (Fetal hemoglobin, Glycophorin A, CD71)
Mycoplasma contamination	all cell lines used in this study tested negative for mycoplasma contamination (PCR detection method)

Commonly misidentified lines (See ICLAC register)	no commonly misidentified cell lines were used in the study.

Animals and other organisms

Policy information about studies involving animals; ARRIVE guidelines recommended for reporting animal research

Laboratory animals	NOD.Cg-PrkdcscidIl2rgtm1Wjl/SzJ (NSG). Human engraftment experimental design and mouse handling followed approved French and European legislation on animal experimentation.<br>NSG females mice were engrafted when 6-8 weeks and euthanized after 16 weeks.
Wild animals	no wild animals were used in the study.
Field-collected samples	no field collected samples were used in the study.
Ethics oversight	This study was approved by ethical committee CEEA-51 and conducted according to French and European legislation on animal experimentation (APAFiS#16499-2018071809263257_v4).

Note that full information on the approval of the study protocol must also be provided in the manuscript.

Flow Cytometry

Plots

Confirm that:

- [x] The axis labels state the marker and fluorochrome used (e.g. CD4-FITC).
- [x] The axis scales are clearly visible. Include numbers along axes only for bottom left plot of group (a 'group' is an analysis of identical markers).
- [x] All plots are contour plots with outliers or pseudocolor plots.
- [x] A numerical value for number of cells or percentage (with statistics) is provided.

Methodology

Sample preparation	Cells were fixed and/or permeabilized using Cytofix/Cytoperm™ (BD Bioscience, San Jose, CA, USA) according to manufacturer's instructions. For live cells analysis, viability was assessed using Zombie Yellow dye (BioLegend, San Diego, CA, USA) as per manufacturers' instructions to exclude dead cells from the analysis. Negative controls were obtained by staining cells only with the isotype control antibodies. For engraftment studies, an Fc Receptor Binding Inhibitor antibody was used to block unspecific binding of mouse Ab to human cells, as per manufacturers' instructions
Instrument	CytoFLEX S, SP6800 Spectral Analyzer, MoFlo cell sorter
Software	Cytexpert, FlowJo (TreeStar)
Cell population abundance	The purity of sorted cells was always controlled post-sorting and >95%; purity was calculated on the "total cell" gate (FSC vs SSC).
Gating strategy	Gating strategy is reported in supplementary info.

- [x] Tick this box to confirm that a figure exemplifying the gating strategy is provided in the Supplementary Information.
